# Supplementary material for: Population structure of Apodemus flavicollis and comparison to Apodemus sylvaticus in northern Poland based on RAD-seq
Source: BMC Genomics. 2020 Mar 18;21:241. doi: 10.1186/s12864-020-6603-3 (PMC7079423; doi:10.1186/s12864-020-6603-3)
Supplement: Supplementary file 1 — Additional file 1 Supplementary materials. [file 12864_2020_6603_MOESM1_ESM.pdf]

Supplementary Material: Population structure of *Apodemus flavicollis* and comparison to *Apodemus sylvaticus* in northern Poland based on RAD-seq

1 Sample information

Table 1: Sample ID, coordinates and environmental information for the samples. Bory - Bory Tucholskie, Bial - Białowieża, Hack - Haćki, Kadz - Kadzidło

ID	Species	Location	Latitude	Longitude	Environment
D04	A. flavicollis	Bory	17.58	53.77	mesic pine forest
E04	A. flavicollis	Bory	17.56	53.81	mesic pine forest
F04	A. flavicollis	Bory	17.55	53.79	mesic pine forest
G04	A. flavicollis	Bory	17.58	53.78	dry pine forest
H04	A. flavicollis	Bory	17.58	53.77	mesic pine forest
A05	A. flavicollis	Bory	17.58	53.77	mesic pine forest
B05	A. flavicollis	Bory	17.58	53.77	sedge meadow
C05	A. flavicollis	Bory	17.58	53.77	mesic pine forest
D05	A. flavicollis	Bory	17.58	53.77	mesic pine forest
E05	A. flavicollis	Bory	17.51	53.80	oak-lime-hornbeam forest
F05	A. flavicollis	Bory	17.58	53.77	mesic pine forest
G05	A. flavicollis	Bory	17.51	53.80	oak-lime-hornbeam forest
H05	A. flavicollis	Bory	17.51	53.80	oak-lime-hornbeam forest
G12	A. flavicollis	Bory	17.51	53.80	oak-lime-hornbeam forest
H12	A. flavicollis	Bory	17.56	53.81	alder foorest at lake
C06	A. flavicollis	Bory	17.58	53.78	reeds at lake

Sample	Species	Location	Long	Lat	Environment
D06	A. flavicollis	Bory	17.51	53.80	oak-lime-hornbeam forest
E06	A. flavicollis	Bory	17.51	53.80	oak-lime-hornbeam forest
F06	A. flavicollis	Bory	17.51	53.80	oak-lime-hornbeam forest
H06	A. flavicollis	Bory	17.56	53.81	mesic pine forest
A07	A. flavicollis	Bory	17.56	53.81	alder fooorest at lake
B07	A. flavicollis	Bory	17.58	53.78	mesic pine forest
C07	A. flavicollis	Bory	17.51	53.80	oak-lime-hornbeam forest
A04	A. flavicollis	Bial	23.85	52.71	cultivated meadow
B04	A. flavicollis	Bial	23.85	52.71	cultivated meadow
C04	A. flavicollis	Bial	23.85	52.71	cultivated meadow
F08	A. flavicollis	Bial	23.83	52.72	oak-lime-hornbeam forest
G08	A. flavicollis	Bial	23.83	52.72	oak-lime-hornbeam forest
H08	A. flavicollis	Bial	23.83	52.72	oak-lime-hornbeam forest
A09	A. flavicollis	Bial	23.83	52.72	oak-lime-hornbeam forest
B09	A. flavicollis	Bial	23.83	52.72	oak-lime-hornbeam forest
C09	A. flavicollis	Bial	23.83	52.72	oak-lime-hornbeam forest
D09	A. flavicollis	Bial	23.83	52.72	oak-lime-hornbeam forest
E09	A. flavicollis	Bial	23.83	52.72	oak-lime-hornbeam forest
F09	A. flavicollis	Bial	23.83	52.72	oak-lime-hornbeam forest
G09	A. flavicollis	Bial	23.83	52.72	oak-lime-hornbeam forest
H09	A. flavicollis	Bial	23.83	52.72	oak-lime-hornbeam forest
A10	A. flavicollis	Bial	23.83	52.72	oak-lime-hornbeam forest
B10	A. flavicollis	Bial	23.83	52.72	oak-lime-hornbeam forest
C10	A. flavicollis	Bial	23.83	52.72	oak-lime-hornbeam forest
D10	A. flavicollis	Bial	23.83	52.72	oak-lime-hornbeam forest
E10	A. flavicollis	Bial	23.83	52.72	oak-lime-hornbeam forest
F10	A. flavicollis	Bial	23.85	52.72	oak-lime-hornbeam forest
G10	A. flavicollis	Bial	23.85	52.72	oak-lime-hornbeam forest
H10	A. flavicollis	Bial	23.82	52.74	oak-lime-hornbeam forest
A11	A. flavicollis	Bial	23.82	52.75	oak-lime-hornbeam forest
B11	A. flavicollis	Bial	23.82	52.70	sedge meadow
C11	A. flavicollis	Bial	23.85	52.72	oak-lime-hornbeam forest
D11	A. flavicollis	Bial	23.85	52.72	oak-lime-hornbeam forest
E11	A. flavicollis	Bial	23.85	52.72	oak-lime-hornbeam forest
F11	A. flavicollis	Bial	23.85	52.72	oak-lime-hornbeam forest

Sample	Species	Location	Long	Lat	Environment
G11	A. flavicollis	Bial	23.85	52.72	oak-lime-hornbeam forest
H11	A. flavicollis	Bial	23.85	52.72	oak-lime-hornbeam forest
A12	A. flavicollis	Bial	23.85	52.72	oak-lime-hornbeam forest
B12	A. flavicollis	Bial	23.85	52.72	oak-lime-hornbeam forest
C12	A. flavicollis	Bial	23.85	52.72	oak-lime-hornbeam forest
D12	A. flavicollis	Bial	23.85	52.72	oak-lime-hornbeam forest
E12	A. flavicollis	Bial	23.85	52.72	oak-lime-hornbeam forest
C02	A. flavicollis	Hack	23.17	52.83	xerothermic meadow
D02	A. flavicollis	Hack	23.17	52.83	xerothermic meadow
E02	A. flavicollis	Hack	23.17	52.83	xerothermic meadow
F02	A. flavicollis	Hack	23.17	52.83	xerothermic meadow
G02	A. flavicollis	Hack	23.17	52.83	xerothermic meadow
H02	A. flavicollis	Hack	23.17	52.83	xerothermic meadow
A03	A. flavicollis	Hack	23.17	52.83	xerothermic meadow
B03	A. flavicollis	Hack	23.17	52.83	xerothermic meadow
C03	A. flavicollis	Hack	23.17	52.83	xerothermic meadow
D03	A. flavicollis	Hack	23.17	52.83	xerothermic meadow
E03	A. flavicollis	Hack	23.17	52.83	xerothermic meadow
F03	A. flavicollis	Hack	23.17	52.83	xerothermic meadow
G03	A. flavicollis	Hack	23.17	52.83	xerothermic meadow
H03	A. flavicollis	Hack	23.17	52.83	xerothermic meadow
D07	A. sylvaticus	Bory	17.54	53.79	dry pine forest
E07	A. sylvaticus	Bory	17.56	53.79	reeds at lake
F07	A. sylvaticus	Bory	17.54	53.79	dry pine forest
G07	A. sylvaticus	Bory	17.55	53.79	mesic pine forest
H07	A. sylvaticus	Bory	17.54	53.79	dry pine forest
A08	A. sylvaticus	Kadz	21.37	53.20	dry pine forest
B08	A. sylvaticus	Kadz	21.37	53.20	dry pine forest
C08	A. sylvaticus	Kadz	21.37	53.20	dry pine forest
D08	A. sylvaticus	Kadz	21.37	53.20	dry pine forest
E08	A. sylvaticus	Kadz	21.37	53.20	dry pine forest

2 Barcodes and demultiplexing

Table 2: Barcodes used and demultiplexing results.

Barcode	Filename	Total	NoRadTag	LowQuality	Retained
TATTCGCAT	D01	1754624	823059	322	802448
CCTTGCCATT	B02	5616138	2656224	1136	2524650
GGTATA	C02	2497516	1178292	412	1112807
TCTTGG	D02	1925168	926463	359	862961
GGTGT	E02	1772988	836727	301	802310
GGATA	F02	2103232	990036	342	950445
CTAAGCA	G02	2396754	1118652	420	1107411
ATTAT	H02	3592492	1672167	603	1654087
GCGCTCA	A03	1701066	796055	316	766110
ACTGCGAT	B03	2859122	1379563	513	1244319
TTCGTT	C03	2522570	1203310	467	1127262
ATATAA	D03	1448256	675350	261	664835
TGGCAACAGA	E03	1907170	896741	415	854373
CTCGTCG	F03	1424136	661282	253	647835
GCCTACCT	G03	1316424	631751	267	579215
CACCA	H03	4119158	1904665	717	1918252
AATTAG	A04	3353928	1576668	531	1528167
GGAACGA	B04	2714032	1268460	499	1237675
ACTGCT	C04	1519814	732180	279	676595
TGCTT	D04	3337318	1598963	538	1516482
GCAAGCCAT	E04	2272530	1077974	436	1028556
CGCACCAATT	F04	1328064	629634	257	597209
CTCGCGG	G04	2843128	1352618	497	1300936
AACTGG	H04	1773388	851270	311	799274
ATGAGCAA	A05	3543298	1701957	692	1580365
CTTGA	B05	2280988	1099255	413	1016552
GCGTCCT	C05	3835930	1834408	674	1724309
ACCAGGA	D05	3081248	1488008	581	1378175
CCACTCA	E05	2003682	940201	332	919846
TCACGGAAG	F05	889424	420138	187	407176
TATCA	G05	1212906	593550	171	545872
TAGCCAA	H05	1794800	838457	312	836413

Barcode	Filename	Total	NoRadTag	LowQuality	Retained
GGTGCACATT	C06	1784198	845365	349	798706
CTCTCGCAT	D06	1495486	710272	290	675749
CAGAGGT	E06	1827948	891153	317	810051
GCGTACAAT	F06	1083614	509870	219	494520
ACGCGCG	G06	1490100	697737	247	686381
GTCGCCT	H06	2562952	1219598	434	1168312
AATAACCAA	A07	2750168	1290585	509	1254852
AATGAACGA	B07	2023934	970073	414	904065
ATGGCAA	C07	2897680	1386103	501	1307711
GAAGCA	D07	4523918	2130884	804	2088074
AACGTGCCT	E07	3561580	1678483	705	1636000
CCTCG	F07	4775646	2243939	795	2212819
CTCAT	G07	2816492	1342690	453	1290660
ACGGTACT	H07	1538254	721448	267	710639
GCGCCG	A08	1581588	751487	306	715924
CAAGT	B08	2362354	1126323	386	1076036
GGAGTCAAG	C08	1931910	921128	340	866712
TGAAT	D08	2004632	977907	329	903723
CATAT	E08	2845620	1348639	463	1306750
GTGACACAT	F08	1793344	840977	320	798641
TATGT	G08	1912488	889366	326	892469
TGCAGA	H08	1587072	744356	247	728265
CATCTGCCG	A09	1927106	894811	400	865134
GGACAG	B09	2391890	1139966	395	1084539
ATCTGT	C09	4006790	1882256	717	1829175
AAGACGCT	D09	2083594	1008686	376	912137
GAATGCAATA	E09	1673516	809802	316	720740
TAGCAG	F09	1611016	772260	268	720206
CTTAG	G09	1236082	639672	195	503807
TTATTACAT	H09	903066	480838	156	346810
GCCAACAAGA	A10	2280156	1095405	452	1002024
TGCCGCAT	B10	4328430	2092480	779	1906904
CGTGTCA	C10	2174200	1069375	366	944630
CAACCACACA	D10	1994002	989286	367	844802
GCTCCGA	E10	2269544	1072489	435	1027809

Barcode	Filename	Total	NoRadTag	LowQuality	Retained
CGTTCA	F10	2396728	1134028	402	1063780
CATCACAAG	G10	1130460	547376	213	482972
TCCAG	H10	1134466	543467	172	500967
AACTGAAG	A11	2060310	972421	383	912133
GATTCA	B11	1559246	726131	226	712556
CAAGCCAATT	C11	2759210	1374653	491	1161521
TTGCGCT	D11	2013912	958443	341	907508
CGCAGACACT	E11	1773052	884978	334	742494
TGTGGA	F11	1638142	778419	287	738748
TGGATA	G11	2001520	982084	343	878640
ATAGCGT	H11	1929208	896385	353	888463
CCATAGA	A12	4910032	2303281	844	2177377
GGCACGCAT	B12	5959610	2968959	1184	2473214
ATTAACAATT	C12	1040872	496149	188	452788
CAATA	D12	2431454	1185404	393	1053547
TAGTCCAT	E12	1326952	615885	252	615685
CGTGACCT	F12	1389748	657705	274	614681
CTTCAGA	G12	9249428	4445105	1627	4157586
ATCTGCAACA	H12	3268002	1557924	623	1457762
	**total**	**206744014**	**98568584**	**36987**	**92741120**
	**min**	**889424**	**420138**	**156**	**346810**
	**max**	**9249428**	**4445105**	**1627**	**4157586**
	**average**	**2404000.16**	**1146146.32**	**430.08**	**1078385.11**
	**stdev**	**1282551.13**	**613923.28**	**234.71**	**575871.53**
	**median**	**2009272**	**975164**	**366.5**	**905786.5**

3 Estimation of the best parameters for the combined dataset

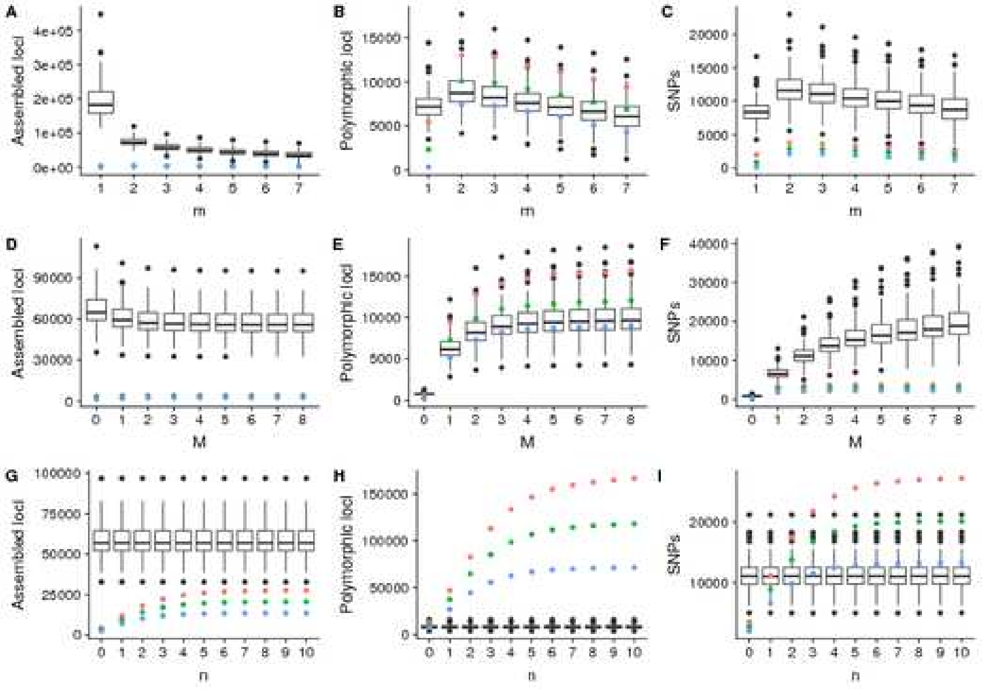

Figure 1: Selection of the optimal parameters for the combined dataset. Number of assembled loci, polymorphic loci and SNPs for iterating values of m, M and n parameters. Blue circles represent data found in at least 40% of the population, green circles in the 60% and red circles in the 80%. Data used to build the figure is available on GitHub: `https://github.com/Marisa89/ddRADseq_poland/blob/master/Tables/Apodemus/Table_selection_best`

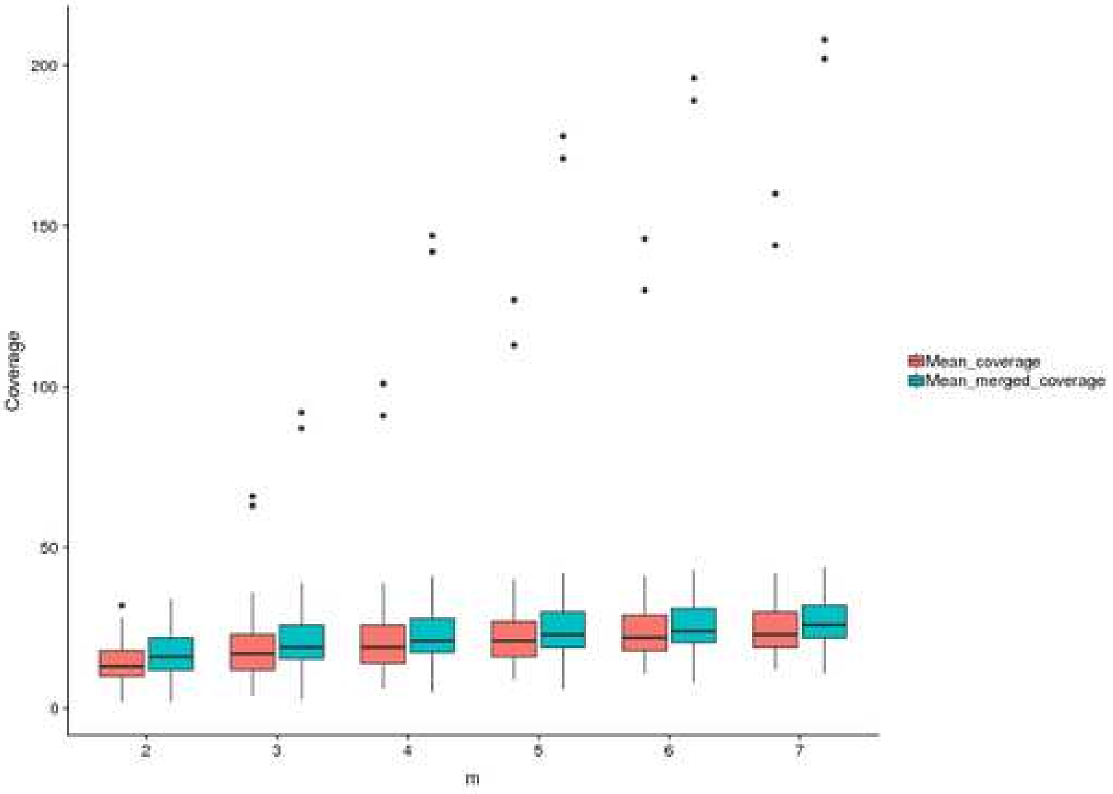

Figure 2: .

Distribution of the mean coverage before and after merging loci for each iteration of the m parameter.Results for the combined dataset including samples from *Apodemus flavicollis* and *Apodemus sylvaticus*. Data used to build the figure is available on GitHub: `https://github.com/Marisa89/ddRADseq_poland/blob/master/Tables/Apodemus/Table_coverage_Apode`

4 Estimation of the best parameters for *Apodemus flavicollis* samples

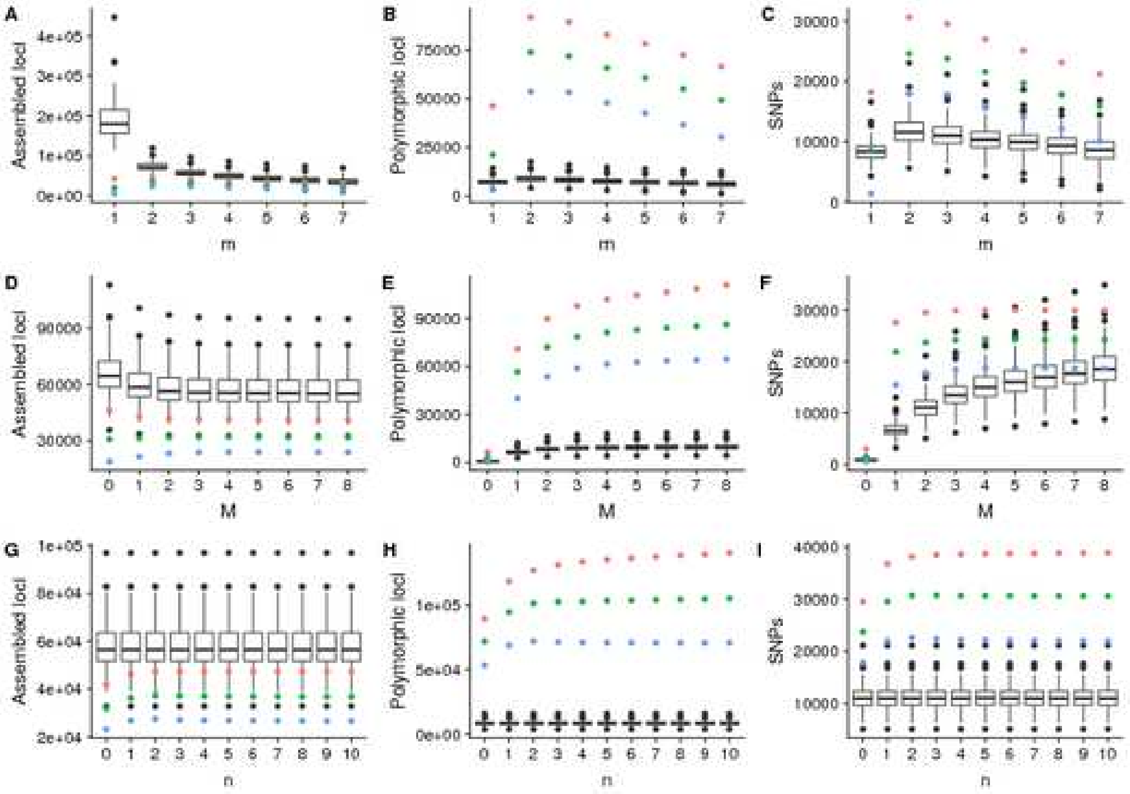

Figure 3: Number of assembled loci, polymorphic loci and SNPs for iterating values of m, M and n parameters. Blue circles represent data found in at least 40% of the individuals, green circles in the 60% and red circles in the 80%. Data used to build the figure is available on GitHub: `https://github.com/Marisa89/ddRADseq_poland/blob/master/Tables/A.flavicollis/Table_selection`

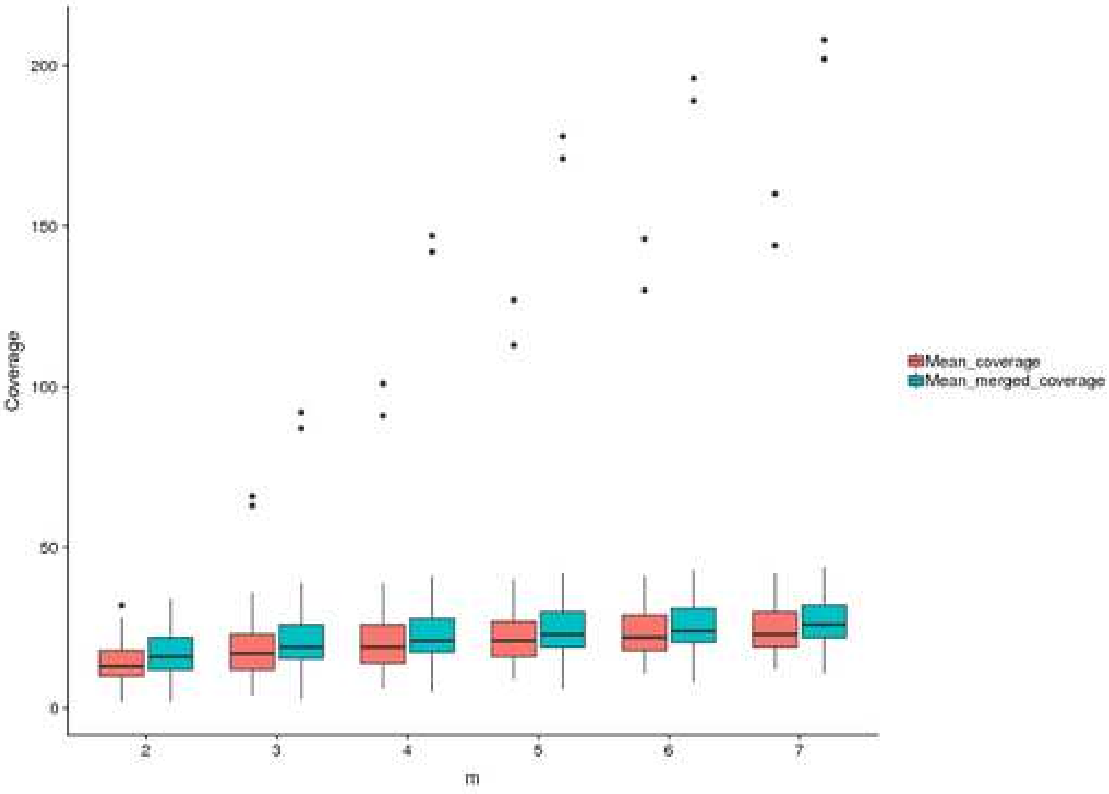

Figure 4: Distribution of the mean coverage before and after merging loci for each iteration of the m parameter for *Apodemus flavicollis* samples. Data used to build the figure is available on GitHub: `https://github.com/Marisa89/ddRADseq_poland/blob/master/Tables/A.flavicollis/Table_coverage_`

5 Cross-validation errors

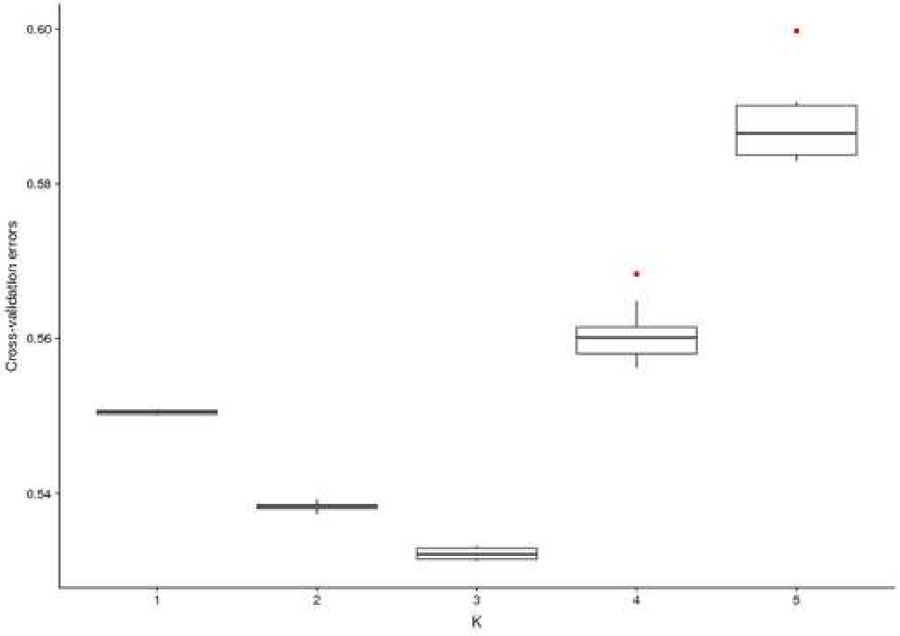

Figure 5: Cross-validation errors obtained for values of K between 1 and 5 for 10 runs with different seeds for all samples

6 Catalogue of loci used for species differentiation

Due to the size of the catalogue, the files has been uploaded into Dropbox. They are available for download at the following link: `https://www.dropbox.com/sh/3757wzer94eef85/AADRXN6GT5`

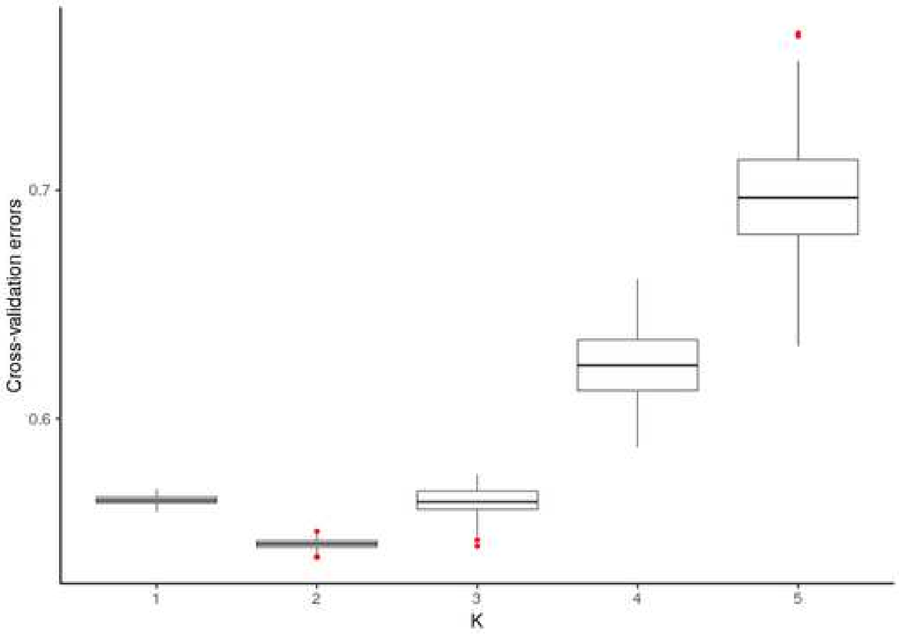

Figure 6: Cross-validation errors obtained for values of K between 1 and 5 for the 100 permutations performed with randomly-drawn equal number of samples per population (n = 15)

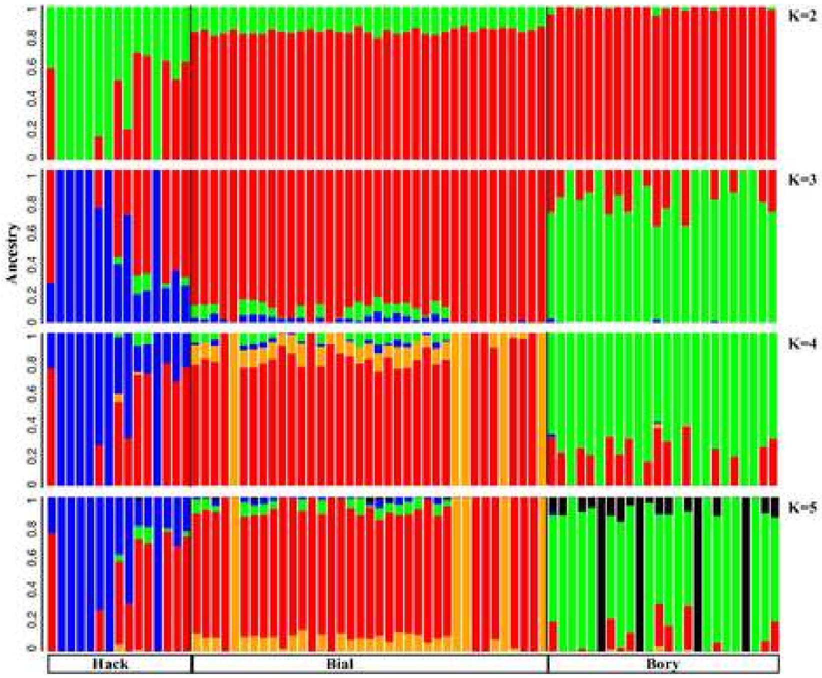

Figure 7: ADMIXTURE plot for K = 2 to K = 5 for all samples

7 117 loci with the highest divergence

Table 3: List of 117 loci with the highest divergence between both species

List of loci with a divergence higher than 4.9%					
3211	11103	20032	35338	45028	62435
4189	11112	20410	35417	45908	62495
4759	12321	20426	35799	47463	62544
4835	12823	21475	36256	51367	62719
4967	13690	22268	36342	51435	62846
5241	13708	23146	36597	51533	64055
5937	13820	23682	36821	53072	64057
6024	14596	24277	37171	53520	64228
6497	14916	25086	37193	53551	64457
6678	15177	25874	38518	53831	64631
7484	15553	26440	39788	54014	65038
7873	16614	26520	39844	57051	65147
8108	16806	27415	39936	57466	65161
8225	17192	30030	40266	59850	65163
9035	17594	31857	40440	60100	66267
9762	18137	32033	41161	60367	66602
10097	18207	32483	42388	60452	67679
10594	19036	32926	42581	61260	
10967	19729	33371	42639	61310	
11041	19799	33510	42900	62087	

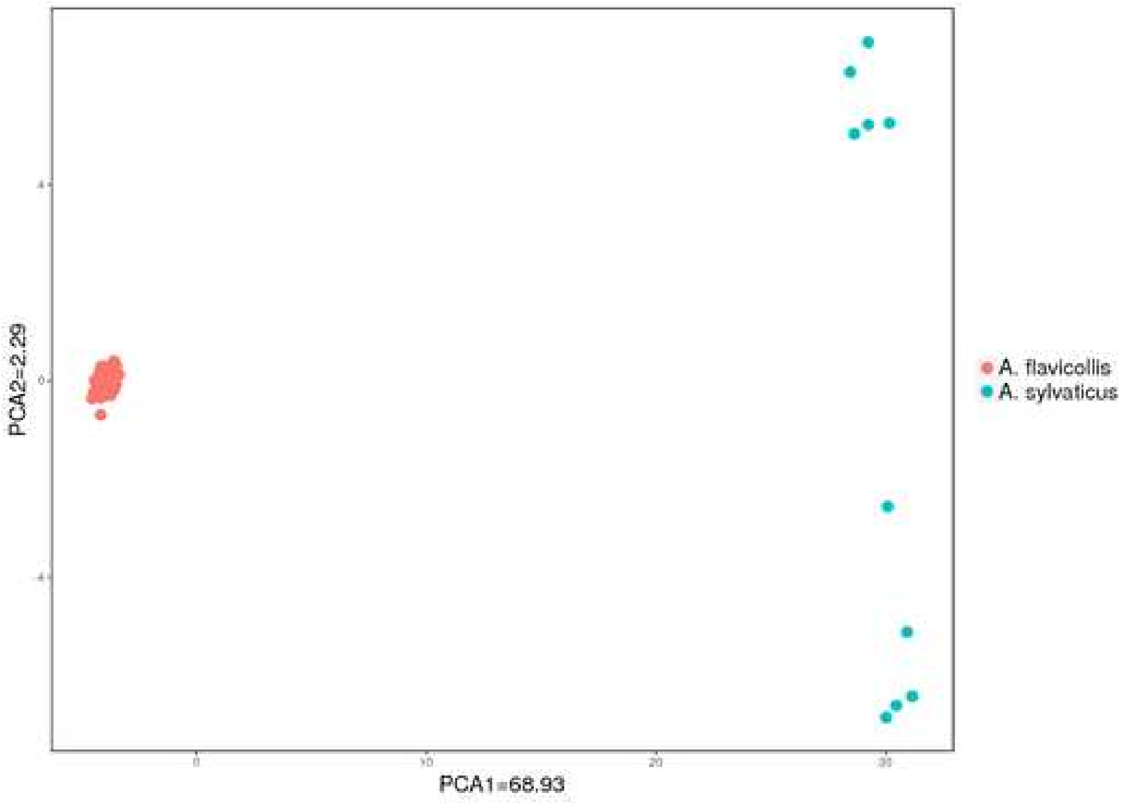

Figure 8: Principal Component Analysis using only the 117 loci with the highest divergence to differentiate Polish samples of *A. flavicollis* and *A. sylvaticus*

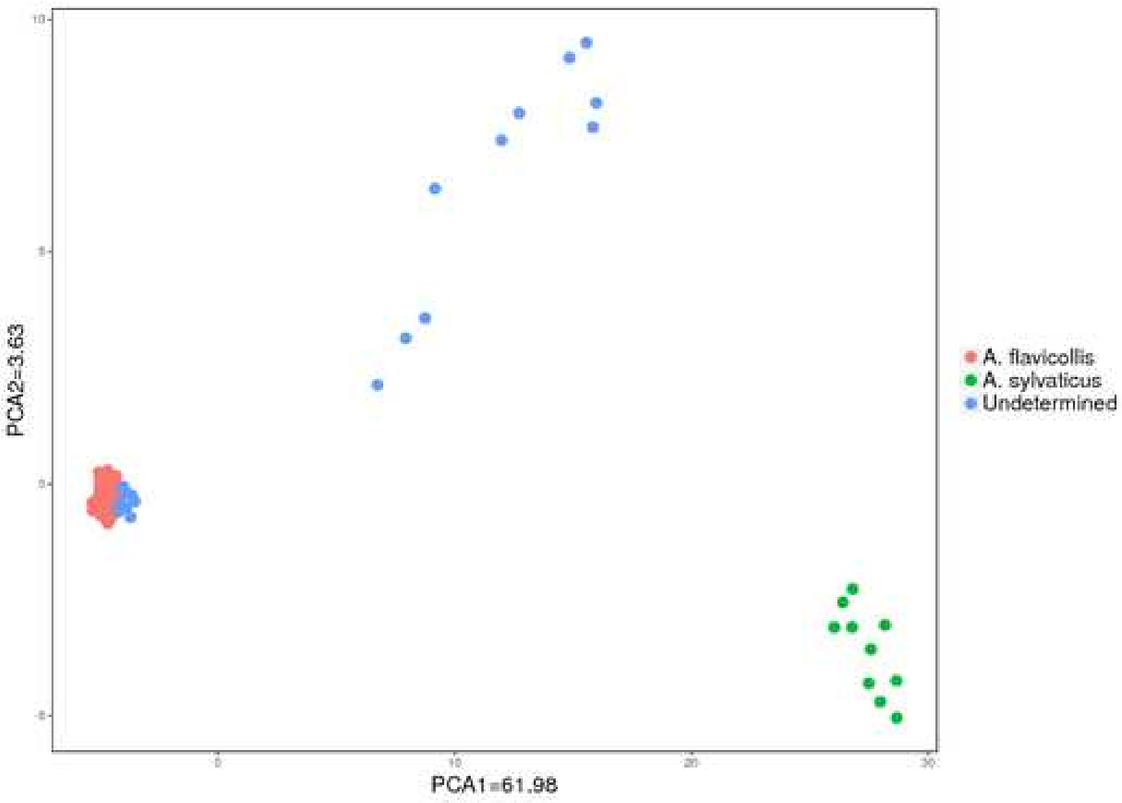

Figure 9: Principal Component Analysis using only the 117 loci with the highest divergence for the dataset including all Polish as well as other European and Tunisian samples.

8 European and Tunisian samples

Table 4: Details of the 20 European and Tunisian samples used to check the catalogue of loci generated based on the Polish samples.

ID	Source	Code
AT1	Johan Michaux	JRM-203
AT2	Johan Michaux	JRM-204
LT1	Karol Zub	JB-466
LT2	Karol Zub	JB-468
LT3	Karol Zub	JB-470
LT4	Karol Zub	JB-485
LT5	Karol Zub	JB-475
RO1	Johan Michaux	JRM-2729
RO2	Johan Michaux	JRM-2720
RO3	Johan Michaux	JRM-2721
WL1	National Museums Scotland	NMS.Z.2009.101.1295M
WL2	National Museums Scotland	NMS.Z.2009.101.1296M
WL3	National Museums Scotland	NMS.Z.2009.101.1203M
WL4	National Museums Scotland	NMS.Z.2009.101.1294M
TN1	Johan Michaux	JRM-138
TN2	Johan Michaux	JRM-139
TN3	Johan Michaux	JRM-140
SC1	National Museums Scotland	NMS.Z.2009.101.1M
SC2	National Museums Scotland	NMS.Z.2009.101.2M
SC3	National Museums Scotland	NMS.Z.2009.101.3M

9 Code

Scripts are available in the following repositories at GitHub:

`https://github.com/Marisa89/ddRADseq_poland/tree/master/Code` 1- Demultiplex_concatenation.sh
2- Iteration_parameter_selection.sh
3- Graphs_Iteration_parameters.R
4- PCA_plots_species.R
5- PCA_plots_flavicollis.R
6- Generate_files_for_divergence.sh
7- SNP_error_rate.sh
8- Loci_Allele_error_rate.sh
9- Allele_error_rate.R
10- Permutations.sh
11- Permutations_genetic_diversity_and_Fst_tables.R
12- Admixture_different_seed.sh

The code used to calculate divergence is available at: `https://github.com/jarekbryk/divergenceR`
